# Fulminant immune checkpoint inhibitor-associated myositis presenting with concurrent myocardial injury and secondary cardiogenic shock in a patient with thymoma: a case report and literature review

**DOI:** 10.3389/fcvm.2026.1909262

**Published:** 2026-09-10

**Authors:** Min Liu, Jiaxin Hu, Ziyu Zheng, Jiayi Chen, Meiru Chen, Zhaohui Zhang, Yayuan Tan

**Affiliations:** 1 Department of Emergency and Critical Care Medicine, the First Clinical Medical College of China Three Gorges University, Yichang Central People's Hospital, Yichang, China; 2 Department of Nursing, School of Health Sciences, China Three Gorges University, Yichang, Hubei, China

**Keywords:** immune checkpoint inhibitors, immune-related adverse events, myocardial injury, myositis, pembrolizumab, thymoma, VA-ECMO

## Abstract

Immune checkpoint inhibitor (ICI)-associated toxicities are uncommon but may rapidly progress to cardiogenic shock and death. Patients with thymoma are considered particularly vulnerable to severe immune-related adverse events because thymic malignancy is closely associated with impaired central immune tolerance and autoimmune dysregulation. We report a 34-year-old man with stage IV type B2 thymoma who developed initial myalgia and fatigue approximately 15 days after the most recent pembrolizumab-containing treatment cycle and progressed to fulminant ICI-associated myositis with respiratory failure and secondary cardiogenic shock by day 19. He presented with severe immune-mediated myositis, concurrent myocardial injury, left ventricular systolic dysfunction, cardiogenic shock, metabolic acidosis, acute kidney injury, acute liver injury, coagulopathy, and respiratory failure. Coronary angiography excluded obstructive coronary disease, and microbiological and virological investigations did not identify an infectious cause. Endomyocardial biopsy and cardiac magnetic resonance imaging were not feasible because of profound hemodynamic instability, VA-ECMO support, and coagulopathy. The patient was treated with early venoarterial extracorporeal membrane oxygenation (VA-ECMO), high-dose methylprednisolone, intravenous immunoglobulin, continuous renal replacement therapy, mechanical ventilation, and comprehensive organ support. Cardiac function improved from a left ventricular ejection fraction of 25% to 51%, VA-ECMO was discontinued on hospital day 7, and the patient survived to discharge. This case and a focused literature review suggest that thymoma-associated ICI myositis with secondary cardiac dysfunction may represent a high-risk phenotype characterized by early onset, overlap neuromuscular toxicity, severe hemodynamic compromise, and potential reversibility when early immunosuppression and advanced circulatory support are implemented.

## 1 Introduction

Immune checkpoint inhibitors (ICIs) targeting the programmed cell death protein 1 (PD-1) and its ligand (PD-L1) have transformed the treatment landscape for multiple malignancies. However, enhanced antitumor immunity may also disrupt peripheral immune tolerance and precipitate immune-related adverse events (irAEs) across virtually all organ systems (1). Among these, ICI-associated myocarditis is uncommon but carries the highest case-fatality rate of all ICI-related toxicities, with reported mortality of 25%–50% in clinically symptomatic patients (2, 3). Importantly, cardiac toxicity frequently coexists with neuromuscular irAEs—particularly myositis and myasthenia gravis—forming a high-mortality overlap syndrome that has been proposed to arise from cross-reactive T-cell responses targeting shared epitopes in skeletal and cardiac muscle (4, 5).

Patients with thymoma face a uniquely elevated risk of severe ICI-related toxicity. Thymic epithelial tumors are characterized by impaired thymic function and defective negative selection of self-reactive T cells, establishing a baseline state of central immune dysregulation that predisposes to autoimmunity—most commonly myasthenia gravis, but also myositis and other autoimmune disorders (6). When ICIs are administered in this setting, phase II clinical trials of pembrolizumab for advanced thymic epithelial tumors have reported substantially higher rates of severe irAEs than in other malignancies, with myocarditis occurring in 5%–9% of patients, frequently overlapping with myositis or myasthenia gravis (7–9). These observations have led to recommendations that ICI therapy be used with particular caution in patients with active thymoma (9).

Clinically, the presentation of ICI-induced overlap syndromes is often catastrophic and rapidly involves multiple organ systems (5). Profound immune-mediated myositis, characterized by extensive skeletal muscle necrosis and a systemic inflammatory storm, can rapidly progress to respiratory muscle failure (10). Severe physiological stress, hypoxia and rhabdomyolysis observed in this clinical setting may be associated with cardiac dysfunction and secondary acute heart failure, as illustrated by reports of takotsubo-like syndrome among ICI-treated patients (11). In acute care settings, managing this complex cascade of neuromuscular and secondary cardiovascular collapse is difficult. The clinical scenario is further complicated when profound hemodynamic instability, coagulopathy, or extracorporeal support dependence precludes definitive tissue characterization via endomyocardial biopsy or cardiac magnetic resonance imaging.

Herein, we report a successfully rescued case of fulminant pembrolizumab-associated myositis with concurrent myocardial injury and cardiogenic shock in a patient with metastatic type B2 thymoma, occurring 15 days after a single dose of pembrolizumab. We systematically review the relevant literature to characterize the clinical phenotype, diagnostic challenges, and therapeutic implications of this high-mortality overlap syndrome, and share a pragmatic, phenotype-based approach to its critical care management.

## 2 Case presentation

### 2.1 Patient information and oncologic history

A 34-year-old man with metastatic thymoma was admitted to the intensive care unit because of rapidly progressive cardiogenic shock and respiratory failure after pembrolizumab-containing treatment. He had been diagnosed with stage IV thymoma (World Health Organization type B2 with pleural metastasis) in September 2024. The patient received his first cycle of chemotherapy (nedaplatin 100 mg and docetaxel 100 mg) on October 8, 2024. During the second cycle on October 29, 2024, pembrolizumab (200 mg) was introduced for the first time in combination with the same chemotherapy regimen. Therefore, the patient received only a single dose (200 mg) of pembrolizumab in total prior to symptom onset. He had no previous history of cardiovascular disease, autoimmune disease, or chronic neuromuscular disorder.

Fifteen days after the most recent pembrolizumab administration, he developed generalized myalgia and fatigue, accompanied by poor appetite and oliguria. Four days later (day 19), rapidly worsening dyspnea and chest tightness developed. Coronary angiography at a local hospital excluded acute coronary syndrome. Because of progressive circulatory collapse and a preliminary clinical suspicion of fulminant myocarditis at the referring hospital, he underwent endotracheal intubation and emergent VA-ECMO support before transfer to our institution.

### 2.2 Initial assessment and diagnostic evaluation

On ICU admission, the patient was deeply sedated and remained dependent on VA-ECMO and vasoactive support. Blood pressure was 89/53 mmHg despite ongoing hemodynamic support, and peripheral extremities were cold and poorly perfused. Initial laboratory evaluation demonstrated severe immune-mediated myositis, concurrent myocardial injury, and systemic hypoperfusion, including creatine kinase (CK) 10,930 IU/L, CK-myocardial band 540 U/L, cardiac troponin I 13 μg/L (reference range: 0–0.04 μg/L), N-terminal pro-B-type natriuretic peptide 12,200 ng/L, arterial pH 7.08, bicarbonate 8.0 mmol/L, base excess −20.56 mmol/L, and lactate 13.87 mmol/L. As the patient presented with fulminant shock and multiorgan failure, early clinical monitoring prioritized markers for shock and rhabdomyolysis; consequently, serial post-admission cTnI measurements were not obtained.

Multi-organ dysfunction was evident. Alanine aminotransferase and aspartate aminotransferase were 1,190 U/L and 1,560 U/L, respectively, serum creatinine increased to 140 μmol/L, and coagulation studies showed severe coagulopathy. In the context of profound shock, the acute liver injury was considered more consistent with systemic hypoperfusion and/or immune-mediated injury than with definite isolated immune-related hepatitis. Inflammatory markers, including C-reactive protein, interleukin-6, and procalcitonin, were elevated. A decreased CD4+/CD8 + ratio (normal reference range: 1.4–2.0) suggested substantial immune perturbation. Subsequent microbiological and virological investigations were negative.

**Figure 1:**
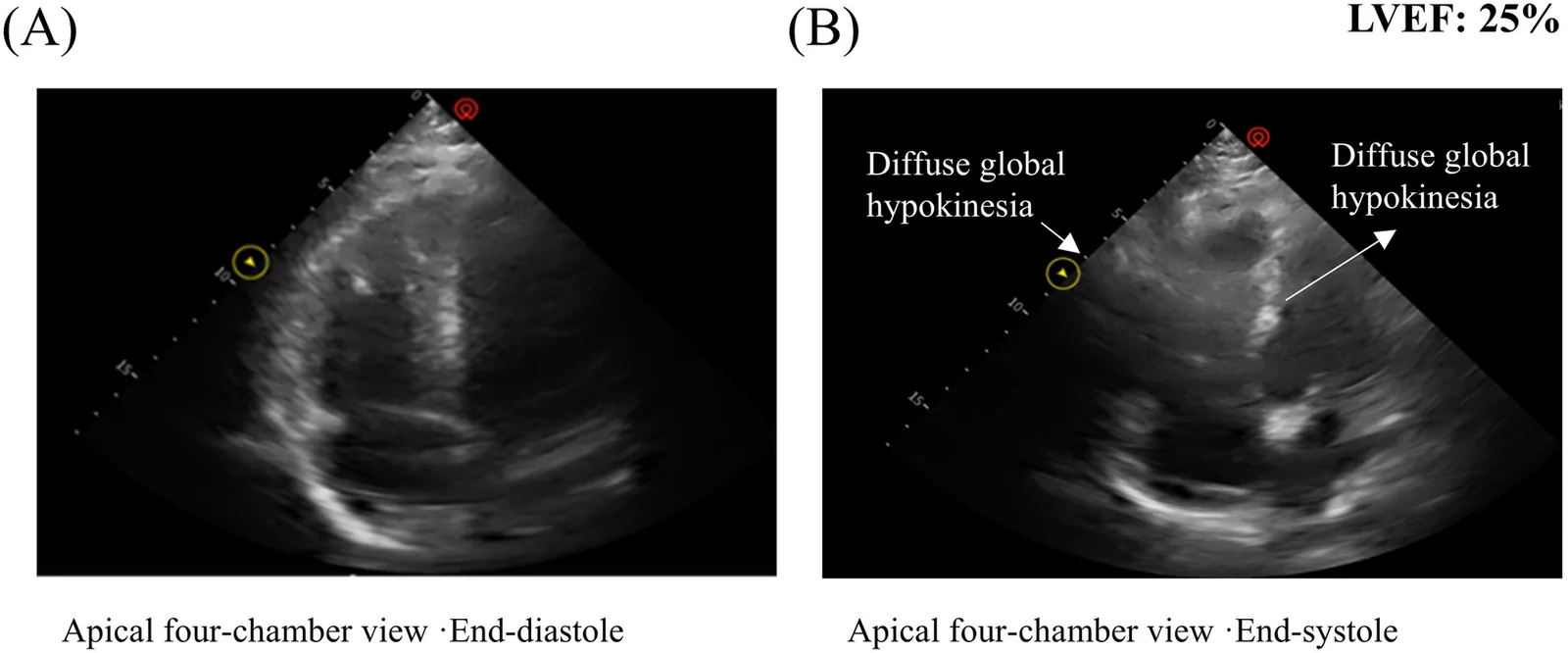
Bedside echocardiography on November 17.

**Figure 2:**
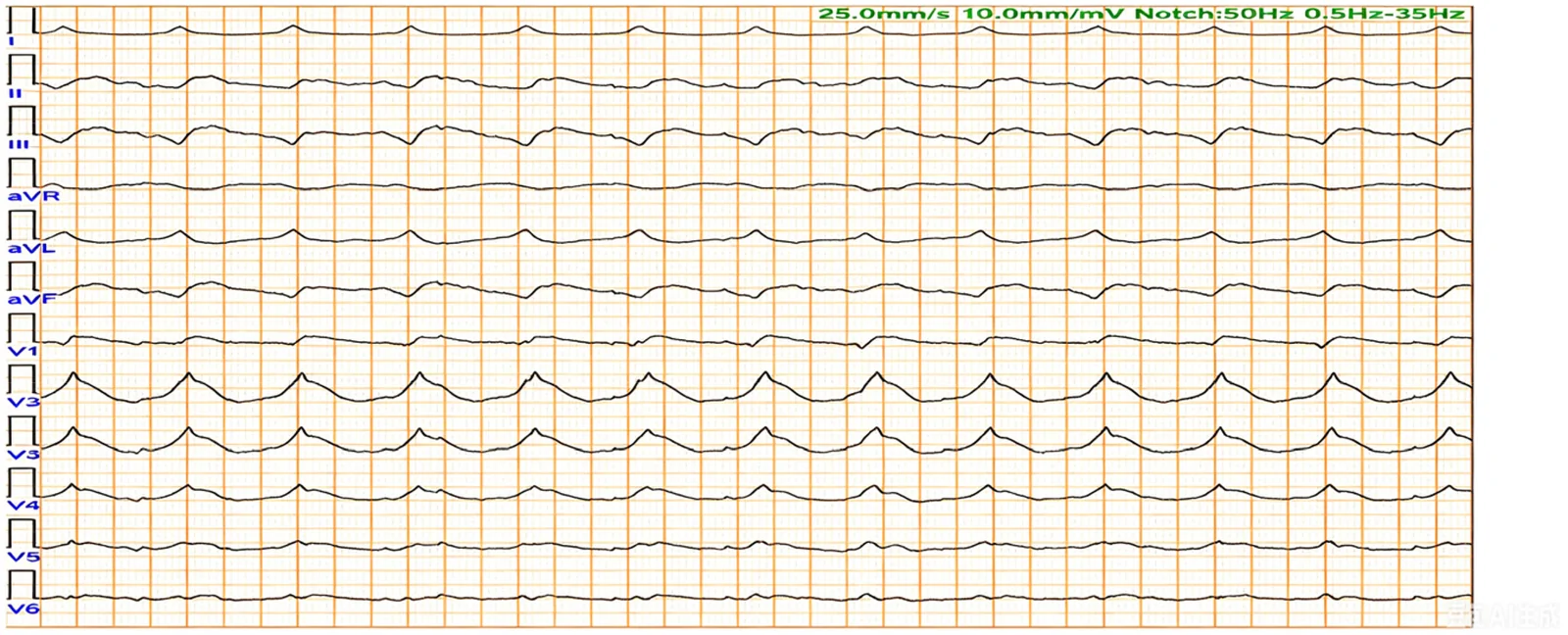
Resting 12-lead electrocardiography on November 18 showed diffuse low QRS voltage in all limb leads (I, II, III, aVR, aVL, aVF) and precordial leads (V1–V6), with ST-segment depression in leads II, III, aVF, and V4–V6 and reciprocal ST-segment elevation in lead aVR.

Transthoracic echocardiography demonstrated severe global left ventricular systolic dysfunction with a left ventricular ejection fraction (LVEF) of 25% and diffuse ventricular hypokinesia (Figure 1). Electrocardiography demonstrated diffuse low QRS voltage and widespread ST-segment abnormalities without diagnostic ST-segment elevation, findings that were compatible with diffuse myocardial injury rather than acute coronary occlusion (Figure 2). Chest radiography and computed tomography demonstrated bilateral pulmonary infiltrates accompanied by small pleural and pericardial effusions, findings compatible with severe cardiopulmonary involvement in the setting of fulminant immune-mediated toxicity. Transient neurological abnormalities, including anisocoria and absent pupillary light reflexes, were observed during profound circulatory shock. Cranial CT and cerebrospinal fluid analysis showed no significant abnormalities, and neurological function recovered completely following hemodynamic stabilization, making primary central nervous system immune-related toxicity unlikely.

Integrating the clinical, laboratory, electrocardiographic, and echocardiographic findings, the case was framed as fulminant immune-mediated myositis with respiratory failure, concurrent myocardial injury, and secondary cardiac dysfunction. This phenotype-based formulation was supported by several converging features: (i) the initial presentation consisted of generalized myalgia and fatigue—skeletal muscle symptoms that preceded respiratory and cardiac deterioration by four days; (ii) the extreme CK elevation (10,930 IU/L); (iii) the presence of concurrent myocardial injury, as evidenced by a marked cTnI elevation (13 μg/L) and new severe global left ventricular systolic dysfunction (LVEF 25%); and (iv) a close temporal relationship with pembrolizumab exposure (symptom onset exactly 15 days after the first and only dose), consistent with ICI-mediated immune toxicity. Acute myocardial infarction was excluded by coronary angiography, and infectious myocarditis or septic shock became less likely after negative microbiological and virological evaluations. Endomyocardial biopsy (EMB) and cardiac magnetic resonance (CMR) imaging were not performed because profound hemodynamic instability, VA-ECMO support, and coagulopathy substantially increased procedural risk and limited safe transfer. In such critical settings, the risk of delaying life-saving immunosuppressive therapy to obtain definitive tissue or imaging confirmation was considered clinically unacceptable. The diagnostic formulation was therefore based on a pragmatic, non-invasive approach integrating recent ICI exposure, the temporal sequence of skeletal muscle versus cardiac symptoms, the magnitude and distribution of biomarker elevation, electrocardiographic abnormalities, new ventricular dysfunction, exclusion of competing causes, and response to immunosuppression.

### 2.3 Therapeutic intervention

A multidisciplinary rescue strategy was initiated immediately after ICU admission. VA-ECMO was maintained to restore systemic perfusion during the acute inflammatory phase of myocardial injury. Initial ECMO parameters included a pump speed of 2,775 rpm and blood flow of 2.23 L/min with 100% oxygen concentration. Severe hemodynamic instability required norepinephrine, metaraminol, dobutamine, and vasopressin to maintain mean arterial pressure above 65 mmHg. Levosimendan was used to support myocardial contractility during recovery, and esmolol was later introduced for rate control after ECMO removal.

Pembrolizumab was permanently discontinued. High-dose intravenous methylprednisolone sodium succinate was initiated at 500 mg/day. Although current guidelines recommend up to 1,000 mg/day for fulminant cases, the 500 mg/day dose was deliberately selected to balance the need for rapid immunosuppression against the extremely high risk of nosocomial infections and bleeding complications associated with concurrent VA-ECMO, severe coagulopathy, and acute liver injury. This was combined with intravenous immunoglobulin (IVIG), which was administered at a dose of 20 g/day for 6 consecutive days (cumulative total dose: 120 g), providing synergistic anti-inflammatory efficacy.

Cardiac biomarkers subsequently declined, and echocardiography showed recovery of LVEF from 25% to 51%, meeting formal VA-ECMO weaning criteria. Consequently, the 500 mg/day intravenous pulse was maintained for 5 days, then abruptly tapered to a reduced maintenance phase of 40 mg/day for 3 days, and permanently discontinued on hospital day 8. No conversion to oral prednisone was performed. This abbreviated, intravenous-only steroid strategy was a highly individualized, risk-adapted decision aimed at minimizing ECMO-related infectious risks. It is important to note that standard guideline-directed slow oral tapering remains the standard of care for severe irAEs to prevent clinical fluctuations and relapses.

**Figure 3:**
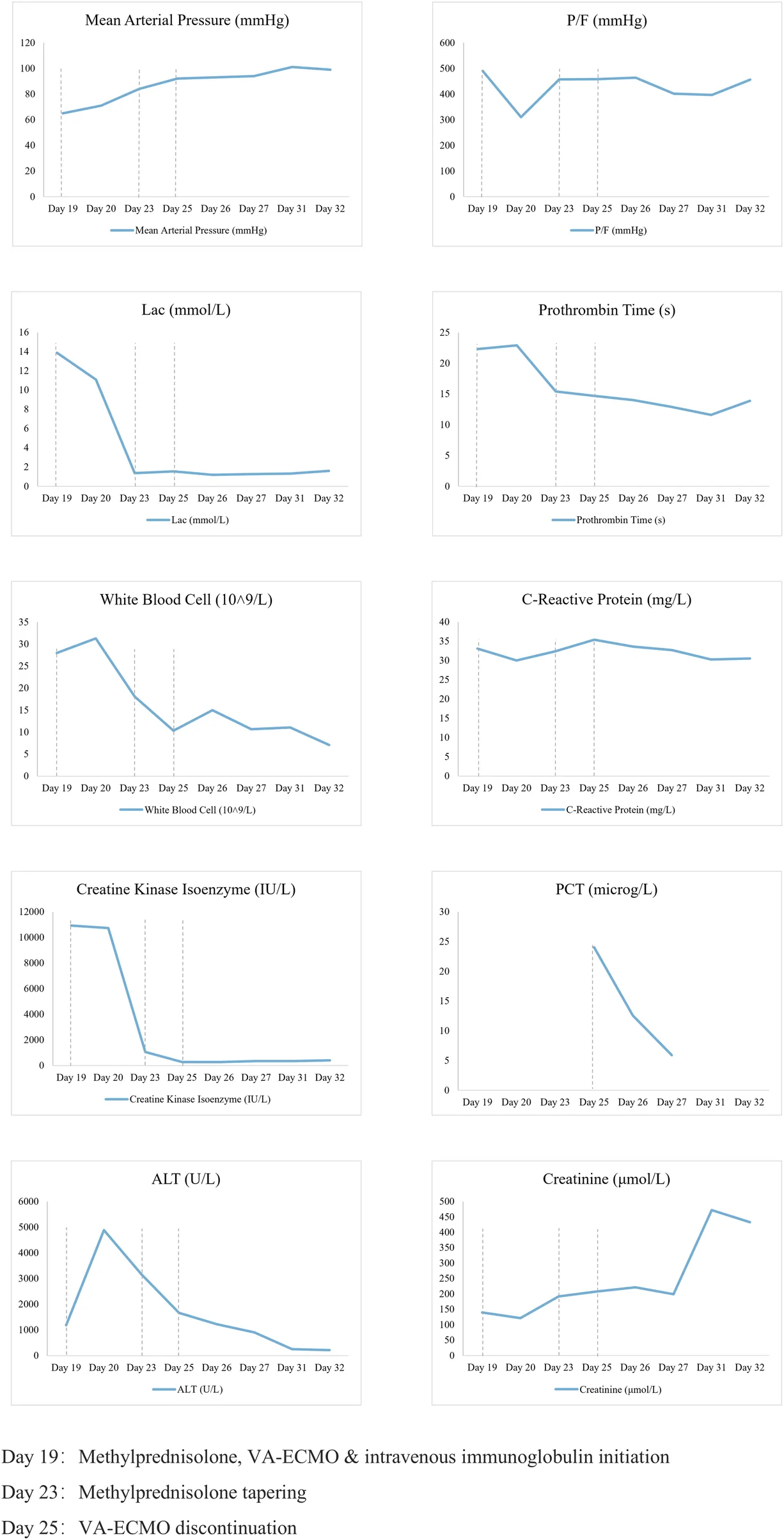
Dynamic changes in the main clinical parameters. Serial post-admission cardiac troponin I (cTnI) was not performed; CK-MB, which is released by both myocardium and skeletal muscle, is presented as a surrogate marker for dynamic tissue injury. Institutional cTnI reference range: 0–0.04 μg/L.

Mechanical ventilation was required during the acute shock phase and was gradually transitioned from controlled ventilation to pressure support ventilation as cardiopulmonary function improved. Continuous renal replacement therapy using the CVVHDF mode was initiated for severe metabolic acidosis, acute kidney injury, hyperlactatemia, and fluid management during circulatory support. Supportive therapy was provided for liver injury, coagulopathy, thrombocytopenia, and metabolic derangements. Empirical broad-spectrum antibiotics were initially administered because inflammatory markers were elevated and the patient was at high risk for secondary infection; cultures remained negative, and antibiotics were discontinued after active infection was excluded. VA-ECMO was successfully discontinued on hospital day 7 after sustained hemodynamic improvement, lactate clearance, recovery of ventricular function, and adequate end-organ perfusion under reduced ECMO flow. Dynamic changes in key clinical parameters are summarized in Figure 3.

### 2.4 Outcome and follow-up

**Figure 4:**
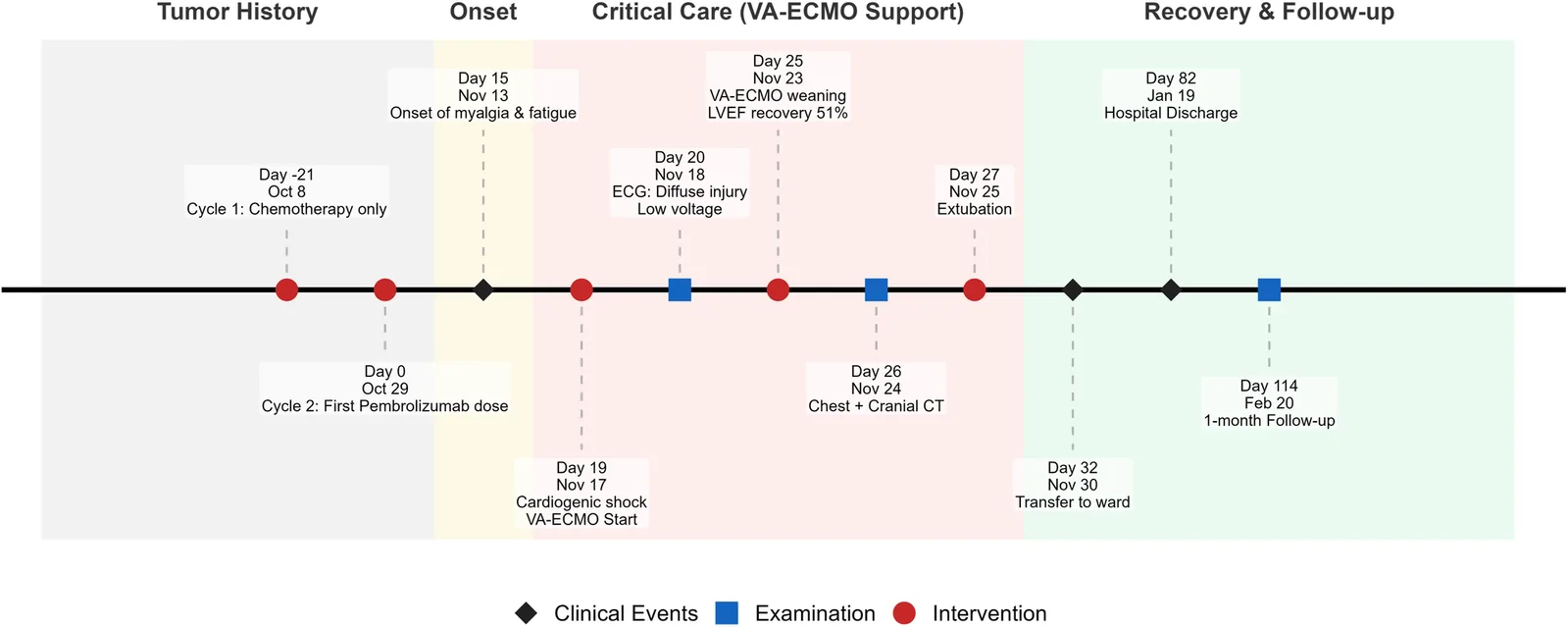
Timeline of the patient's clinical course from cancer diagnosis to follow-up, following CARE guidelines.

The patient demonstrated progressive recovery of cardiac, respiratory, renal, and hepatic function. Lactate normalized, metabolic acidosis resolved, and vasoactive agents were discontinued. He was transferred from the ICU to the general ward on November 30, 2024, and discharged on January 19, 2025, in stable condition. At 1-month follow-up after discharge, he remained alive without recurrence of severe immune-related toxicities. Mild exertional chest tightness and dyspnea persisted, and NT-proBNP remained mildly elevated; continued follow-up through the cardio-oncology clinic was arranged. Post-discharge CMR imaging applying Lake-Louise criteria was strongly recommended to verify myocardial inflammation after clinical stabilization. However, the patient failed to complete this crucial imaging study due to practical barriers, including receiving off-site medical care at external outpatient facilities and personal non-adherence. Consequently, raw external imaging datasets and serial recovery-phase 12-lead electrocardiograms could not be retrieved by our hospital. The overall clinical timeline from cancer diagnosis to follow-up is illustrated in Figure 4.

## 3 Discussion

This case illustrates an aggressive but potentially reversible course of fulminant pembrolizumab-associated myositis presenting with severe cardiac dysfunction in a patient with thymoma. The patient developed nonspecific systemic and muscular symptoms shortly after ICI exposure, followed by rapid progression to severe myocardial injury, cardiogenic shock, metabolic acidosis, respiratory failure, and multi-organ dysfunction. Survival was achieved through early recognition, discontinuation of pembrolizumab, high-dose immunosuppression, VA-ECMO, CRRT, and coordinated critical care support.

The biological plausibility of this presentation is strong, rooted in the disruption of multiple layers of immune tolerance. PD-1 signaling contributes to immune tolerance, including in the myocardium, and pathway blockade may promote autoreactive T-cell activation, myocardial infiltration, and myocyte injury (4, 12–16). In patients with thymic epithelial tumors, this susceptibility may be further amplified by impaired central immune tolerance resulting from defective negative selection of self-reactive T cells within the abnormal thymic microenvironment (6, 17). Consistent with this concept, thymoma itself is strongly associated with autoimmune diseases, particularly myasthenia gravis, and less commonly with inflammatory myopathy and myocarditis, even in the absence of immune checkpoint inhibition (18, 19).

We therefore speculate that PD-1 blockade may function as a “second hit” in this immunologically vulnerable population, further amplifying autoreactive immune responses. Because thymoma is associated with immune responses against striational muscle antigens, including titin and ryanodine receptor-related antigens (19), PD-1 blockade may facilitate immune injury involving both cardiac and skeletal muscle. This mechanism may partly explain the overlap syndrome of myositis, myocarditis, and myasthenia gravis that has been increasingly recognized in patients receiving immune checkpoint inhibitors (2, 20).

Arriving at a definitive diagnosis in this complex scenario presented several clinical challenges. The patient initially developed generalized myalgia coupled with a markedly elevated CK level (10,930 IU/L), strongly suggesting fulminant immune-mediated myositis as the predominant pathology. Given the patient's severe hemodynamic instability, a complete confirmatory work-up, including electromyography, muscle biopsy, and specific autoantibody assays, could not be safely performed. Despite this lack of tissue diagnosis, the clinical and biochemical trajectory was highly consistent with severe muscle toxicity. Consequently, the primary diagnostic challenge centered on characterizing the nature of the concurrent cardiac involvement.

To contextualize this challenge and our patient's phenotype within the existing literature, we performed a comprehensive literature search in PubMed for articles published up to June 5, 2026. The search strategy was designed to capture both ICI-associated cardiovascular and neuromuscular toxicities in thymoma patients using the following string: (Thymoma[MeSH Terms] OR Thymic Neoplasms[MeSH Terms] OR Thymoma[TIAB] OR Thymic[TIAB]) AND (Immune Checkpoint Inhibitors[MeSH Terms] OR checkpoint inhibitor[TIAB] OR pembrolizumab[TIAB] OR nivolumab[TIAB] OR sintilimab[TIAB] OR toripalimab[TIAB] OR tislelizumab[TIAB] OR camrelizumab[TIAB] OR PD-1[TIAB] OR PD-L1[TIAB]) AND (Myocarditis[MeSH Terms] OR myocarditis[TIAB] OR Myositis[MeSH Terms] OR myositis[TIAB] OR rhabdomyolysis[TIAB] OR myasthenia gravis[TIAB] OR cardiotoxicity[TIAB] OR cardiac toxicity[TIAB]). This initial search yielded 86 records. During title and abstract screening, 68 records were excluded (58 lacking individual patient data, 8 involving non-thymoma malignancies and 2 preprints/duplicates). After a full-text review of the remaining 18 articles, 8 were further excluded (7 lacked objective evidence of concurrent myositis, and 1 lacked cardiac involvement). Ultimately, 10 unique publications documenting 11 previously reported cases met our inclusion criteria and were pooled with our index case (21–30) (Table 1).

**Table 1:** Summary of previously published cases and the present case of thymoma-associated immune checkpoint inhibitor myositis (*n* = 12).

Case	Age/Sex	Histology	ICI Agent	Onset (days)	Concomitant irAEs	ECMO Type	Peak Troponin	Peak CK (U/L)	Nadir LVEF (%)	Electrical Abnormalities	Immunosuppression	Survival	Time from symptom onset to immunosuppression	ECMO duration
Konstantina 2019 (21)	30/F	B3	Pembrolizumab	3	Myositis, myocarditis, MG, hepatitis, respiratory failure, septic shock	No	NR	Elevated	NR	Ischemic changes	Steroids + IVIG + Rituximab + Pyridostigmine	Died	Not explicitly reported	Not used
Hyun 2020 (22)	45/F	B2	Pembrolizumab	15	Myositis, myocarditis, MG crisis, hepatitis, cardiorenal syndrome, respiratory failure	No	42 ng/mL cTnI	7,552	28	AV block, bundle branch block, cardiac arrest, VT	Steroid pulse	Died	Approximately 3 days	Not used
Portolés 2021 (23)	48/F	B3	Pembrolizumab	10	Myositis, myocarditis, MG, hepatitis, renal failure, heart failure, respiratory failure	No	13.343 ng/L hs-cTnI	2,430	10	AV block, BBB, escape rhythm, ventricular rhythm	Steroid pulse + Infliximab + ATG + Pyridostigmine	Died	Approximately 3 days	Not used
Luo 2021 (24)	47/F	B2	Toripalimab	28	Myositis, myocarditis, MG, respiratory failure	No	2.796 ng/mL cTnI	25,200	NR	Sinus tachycardia, BBB, AV block, cardiac arrest	Steroids + IVIG	Survived	Approximately 7 days	Not used
Yang 2021 (25)	33/M	NR	Sintilimab	34	Myositis, myocarditis, MG	No	1566 ng/L cTnT	25,692	61	BBB	Steroids + IVIG + Pyridostigmine	Survived	Not explicitly reported	Not used
Jang 2022 (26)	48/F	NR	Pembrolizumab	13	Fulminant myositis, cardiotoxicity (cardiac enzyme elevation), prior MG history	No	NR (elevated, see Figure 1 of original publication)	∼5,500	Normal	T wave inversion	Steroids + MMF + IVIG + PLEX	Survived	Not explicitly reported	Not used
Nguyen 2022 (27)	25/M	NR	Pembrolizumab	14	Myositis, myocarditis, cardiogenic shock	VA-ECMO	3,000 ng/L cTnT	3,714	<10	Sustained VT, intraventricular block	Steroid pulse + MMF + Abatacept + Ruxolitinib	Survived	<24 h	Approximately 7 days
Liu 2022-1 (28)	42/M	B3	Sintilimab	13	Myositis, myocarditis, immune hepatitis, type II respiratory failure	No	12.97 ng/mL hs-cTnI	10,802	60	Sinus tachycardia, BBB, ST-T changes, QTc prolongation, low voltage	Steroid pulse + IVIG	Died	Approximately 3 days	Not used
Liu 2022–2 (28)	52/M	B3	Tislelizumab	21	Myositis, myocarditis, immune hepatitis, acute heart failure, MG relapse	No	18.59 ng/mL hs-cTnI	9,290	NR	BBB, escape rhythm, AV block, intraventricular block, QTc prolongation	Steroid pulse + IVIG + MMF	Survived	Approximately 1 day	Not used
Wang 2023 (29)	45/F	B2	Sintilimab	14	Myositis, myocarditis, MG, arrhythmia storm, cardiogenic shock, heart failure	No	0.3625 ng/mL cTnI	22,535.71	71	QTc prolongation, BBB, T wave changes, ventricular arrhythmias	Steroids + IVIG + PLEX	Died	Not explicitly reported	Not used
Zhao 2025 (30)	53/M	B2	Camrelizumab	14	Myositis, myocarditis, MG	No	4.31 ng/mL cTnI	4,567	71	sinus tachycardia	Steroids + IVIG	Survived	1 day	Not used
Present case 2026	34/M	B2	Pembrolizumab	15	Myositis, myocardial injury, secondary heart failure/cardiogenic shock, acute liver injury, AKI, coagulopathy, MODS	VA-ECMO	13 ng/mL cTnI	10,930	25	Low voltage; widespread ST-segment changes	Steroids + IVIG	Survived	4 days	7 days

Troponin subtypes (cTnI, cTnT, hs-cTnI) and units are retained as reported in the original publications. Direct cross-case comparison of absolute troponin values is not feasible due to differences in detection assays and institutional reference ranges. Onset (days) is defined as the interval from the last infusion of PD-1 inhibitor to the first presentation of immune-related adverse symptoms (myalgia, fatigue, dyspnea, or ocular weakness).

AKI, acute kidney injury; ATG, anti-thymocyte globulin; AV, atrioventricular; BBB, bundle branch block; MG, myasthenia gravis; MMF, mycophenolate mofetil; MODS, multiple organ dysfunction syndrome; NR, not reported; PLEX, plasma exchange; VT, ventricular tachycardia.

As summarized in Table 1, the overlap of immune-mediated myositis and severe cardiac dysfunction in thymoma patients represents a high-risk clinical phenotype. Most cases developed within the first month after ICI exposure (range, 3–34 days), with a median onset of approximately 15 days. Concomitant myasthenia gravis was also frequently observed. Among the 12 pooled cases, 5 patients died, yielding an overall mortality of 41.7%. This estimate, however, must be interpreted with caution. Due to inherent publication and selection biases, this figure should be viewed as an indicator of the severity of this overlap syndrome rather than a true case-fatality rate. Reviewing this cohort also underscores a pervasive diagnostic limitation in the current literature. Among the 12 pooled cases, only one case (Portolés et al., 2021) had endomyocardial biopsy confirmation of lymphocytic myocarditis, and none underwent cardiac magnetic resonance imaging; the remaining 11 cases (91.7%) were diagnosed on clinical grounds alone, without histopathological or tissue-characterization confirmation.

Our index case exemplifies this diagnostic uncertainty. The patient initially presented with generalized myalgia and fatigue—skeletal muscle symptoms that preceded respiratory failure and cardiogenic shock by four days—and the extreme CK elevation (10,930 IU/L) indicates that fulminant immune-mediated myositis was a dominant component of the phenotype. Concurrent myocardial injury was also clearly present, as evidenced by a marked cTnI elevation (13 μg/L; approximately 325-fold the upper limit of normal) and new severe left ventricular systolic dysfunction (LVEF 25%). However, because cTnI was measured only on a single occasion without serial trending, and neither endomyocardial biopsy nor cardiac magnetic resonance could be performed, it is not possible to determine with certainty whether the cardiac dysfunction represented primary ICI-associated myocarditis or myocardial injury occurring within a broader myositis-predominant overlap syndrome. We therefore frame the diagnosis as fulminant immune-mediated myositis with concurrent myocardial injury and secondary cardiac dysfunction, acknowledging that definitive histopathological characterization was not feasible.

Early VA-ECMO was likely central to survival. Mechanical support restored systemic perfusion, reversed shock physiology, and created a therapeutic window during which immunosuppression could control myocardial inflammation. The relatively short ECMO duration and rapid LVEF recovery suggest that early initiation before irreversible myocardial or multi-organ injury may improve reversibility in selected patients with severe ICI-associated cardiotoxicity and myositis. At the same time, this case highlights the need for careful reassessment of infection. Elevated procalcitonin and IL-6 may reflect immune-mediated hyperinflammation rather than true infection; in this patient, cultures were negative and inflammatory markers improved in parallel with immunosuppression. Empirical antibiotics may be reasonable initially in unstable patients, but de-escalation should follow microbiological evidence and clinical reassessment.

Several limitations should be acknowledged. First, EMB and CMR imaging were not performed, precluding histopathological confirmation. Consequently, a definitive attribution of the multi-organ injury solely to pembrolizumab is challenging. Given that the patient received combination therapy with docetaxel and nedaplatin, their potential contribution to the myocardial and multi-organ injury cannot be entirely excluded in the absence of histological confirmation. Second, neurological abnormalities and acute liver injury could not be definitively attributed to immune-related toxicity because critical illness, shock, hypoperfusion, metabolic derangement, and sedative exposure may have contributed. Finally, the literature review was based on published case reports and is subject to reporting bias and incomplete data.

A final important consideration relates to the immunosuppressive regimen. Current guidelines strongly recommend a slow and prolonged tapering of corticosteroids for severe immune-related adverse events, as clinical fluctuations and disease relapses are highly expected in these scenarios. The rapid tapering and complete discontinuation of intravenous steroids within 8 days in our case represents an exceptional, high-risk deviation from standard treatment protocols. This abbreviated regimen was necessitated solely by the patient's extreme risk of catastrophic nosocomial infections and severe coagulopathy while on VA-ECMO. We strongly emphasize that such a rapid taper is not recommended for general practice, and standard guideline-directed slow tapering remains the absolute standard of care to prevent relapses.

## 4 Conclusion

Fulminant pembrolizumab-associated myositis presenting with concurrent myocardial injury and secondary cardiogenic shock in thymoma may progress rapidly from nonspecific myalgia and fatigue to multi-organ dysfunction. This case supports intensified surveillance during the first weeks after ICI initiation in thymoma patients, especially when muscular symptoms or CK elevation occur. Early discontinuation of the offending ICI, prompt high-dose immunosuppression, and timely VA-ECMO may provide a bridge to recovery in selected fulminant cases.

## Data Availability

The original contributions presented in the study are included in the article/Supplementary Material, further inquiries can be directed to the corresponding author.
